# Imaging pediatric osteomyelitis: The role of ^99m^Tc-HDP bone scintigraphy and the additional value of SPECT/CT imaging

**DOI:** 10.1016/j.radcr.2025.11.062

**Published:** 2025-12-13

**Authors:** Saxby Brown, Eva A. Wegner, Monica A. Rossleigh

**Affiliations:** a Department of Nuclear Medicine, Prince of Wales and Sydney Children’s Hospital, Barker Street, Randwick, NSW 2031, Australia; b The University of New South Wales, Faculty of Medicine, Cnr High Street & Botany Street, Kensington, NSW 2033, Australia

**Keywords:** Osteomyelitis, Bone scintigraphy, SPECT/CT, Pediatric, Talar bone, Lumbar spine

## Abstract

Diagnosing pediatric osteomyelitis can be challenging due to its often nonspecific symptoms and the potential for early or misleadingly normal laboratory results. Early and accurate diagnosis is essential to prevent long-term complications. This report discusses 2 cases: an 18-month-old girl with a 4-week history of a worsening right leg limp and refusal to weight-bear, presenting afebrile and with normal inflammatory markers, where triple-phase bone scintigraphy with SPECT/CT was crucial in diagnosing talar osteomyelitis; and a 14-month-old boy presenting with a 5-day history of refusing to walk and mildly raised inflammatory markers, where triple-phase bone scintigraphy with SPECT/CT identified the presence of L5 vertebral osteomyelitis. These 2 cases demonstrate the utility of whole body bone scintigraphy and the additional value in SPECT/CT acquisition in accurately localizing osteomyelitis in children, particularly when other investigations are nondiagnostic or symptoms are not clearly localized, or when MRI is logistically challenging due to the requirement for general anesthesia.

## Introduction

Osteomyelitis remains one of the most prevalent musculoskeletal infections in children and often occurs due to the hematogenous spread of bacteria to bone, with a predilection for the metaphysis due to its high vascularity and susceptibility to trauma [1]. The diagnosis of acute osteomyelitis presents a clinical challenge, as common presenting symptoms such as fever, lethargy, or irritability are nonspecific, and early investigations are often normal [2]. Notably, blood cultures identify the causative organism in only 50% of cases [3]. Early diagnosis and treatment are important to prevent long-term complications, such as chronic infection or growth disturbances [4].

Plain radiography is the standard initial investigation but often fails to definitively diagnose acute osteomyelitis, as bony changes may not be apparent for approximately 14 days [5,6]. Ultrasound and diagnostic CT have specific adjunctive roles; ultrasound offers good sensitivity for superficial collections, while diagnostic CT is useful for detecting later-stage bone destruction [7]. To address the need for early detection, radionuclide bone scintigraphy is frequently utilized due to its high sensitivity. The addition of single-photon emission computed tomography (SPECT) significantly improves diagnostic specificity [8]. This is further enhanced by concurrent low-dose computed tomography (CT), which is obtained for anatomical localization and attenuation correction using a radiation dose specifically optimized to provide structural information without the higher exposure associated with diagnostic CT protocols [9].

While Magnetic Resonance Imaging (MRI) remains the reference standard for detecting early marrow edema and soft-tissue extension, its utility in this age group is frequently compromised by the requirement for absolute immobility [10]. In children aged 1 to 4 years, this often necessitates General Anesthesia (GA) or deep sedation, introducing potential anesthetic risks and placing a significant burden on hospital resources given the limited availability of MRI slots amenable to GA [11]. Consequently, SPECT/CT nuclear imaging offers a valuable diagnostic alternative. This report details 2 cases of pediatric osteomyelitis, in an 18-month-old girl and a 14-month-old boy, where triple-phase bone scintigraphy with SPECT/CT played a crucial role in the diagnosis and localization of osteomyelitis.

### Case report #1

An 18-month-old girl initially presented with a 1-week history of a viral illness and a new-onset right leg limp. A clinical assessment at the time suggested transient ankle joint synovitis. There was no history of trauma. Her family history was notable for hip dysplasia in both paternal aunts. The pregnancy was complicated by gestational diabetes, but the child was born at term with no neonatal complications.

Over the following 3 weeks, her limp worsened, and by the time of her next presentation she was refusing to weight-bear on her right leg. She was afebrile, and the remaining vital signs were within normal limits. Clinical examination revealed a right-sided limp with circumduction of the right leg. She was able to weight-bear intermittently, often on her tiptoes. There was no obvious swelling, erythema, or bruising. Tenderness was noted over the lateral aspect of the right hip, and she resisted internal rotation of the hip. Examination of the knees, ankles, and feet was otherwise unremarkable. Neurological examination was normal.

Initial blood tests on re-presentation showed a normal white cell count (WCC) of 12 × 10^9^/L (normal range 4.5-15 × 10^9^/L) and C-reactive protein (CRP) of 1 mg/L (normal range < 3 mg/L). Blood cultures were negative. An ultrasound of the right foot and ankle showed no significant effusion or hyperemia in the tibiotalar joint, but did reveal a small amount of fluid in Kager's fat pad adjacent to the right posterior ankle, without surrounding soft tissue hyperemia. Plain radiographic imaging of the hips and right lower limb showed no evidence of fracture or osteomyelitis. A nasopharyngeal aspirate was positive for Respiratory Syncytial Virus (RSV).

**Fig. 1:**
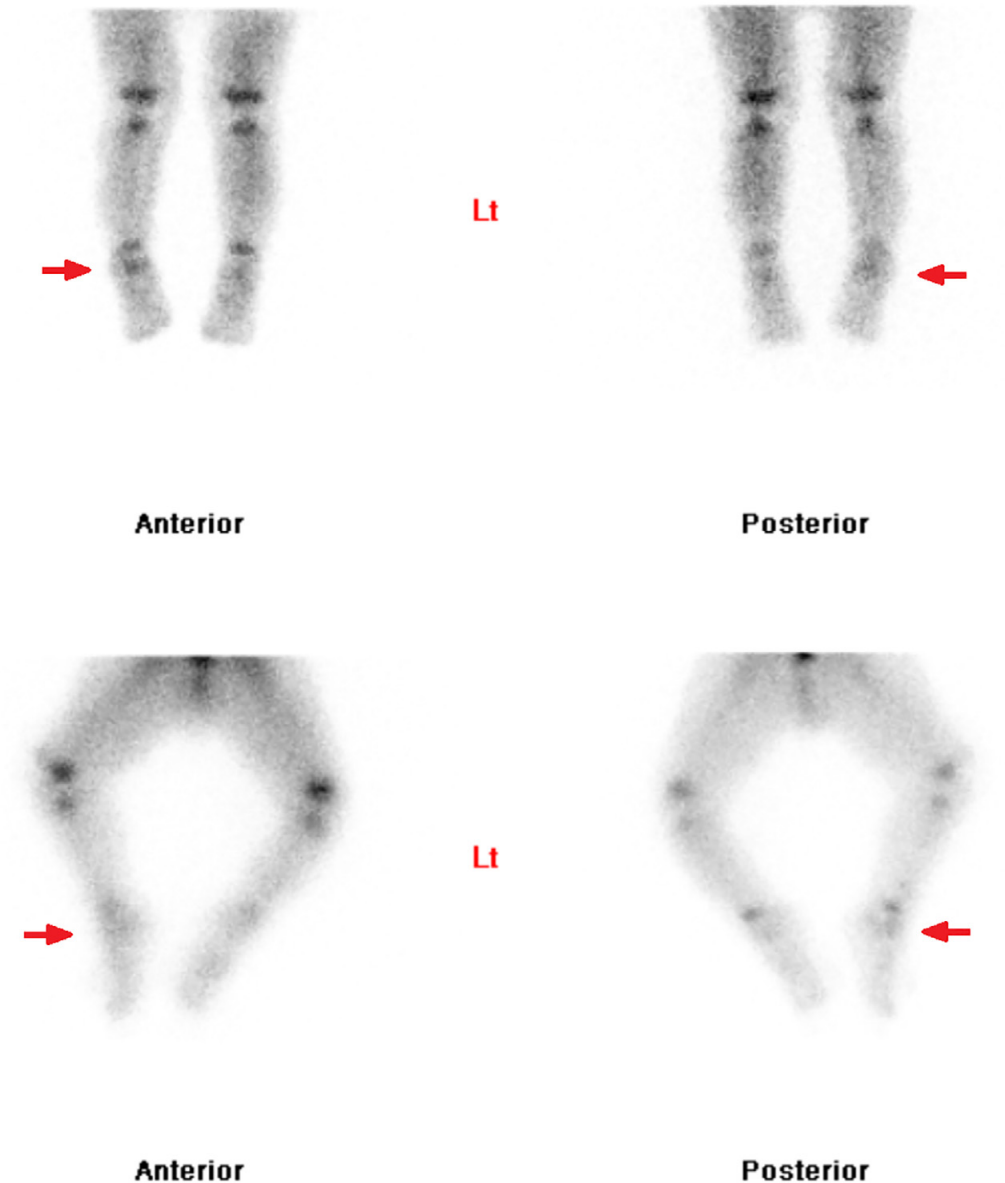
Blood-pool imaging of the lower limbs, showing subtle asymmetrical soft tissue hyperemia at the right ankle.

**Fig. 2:**
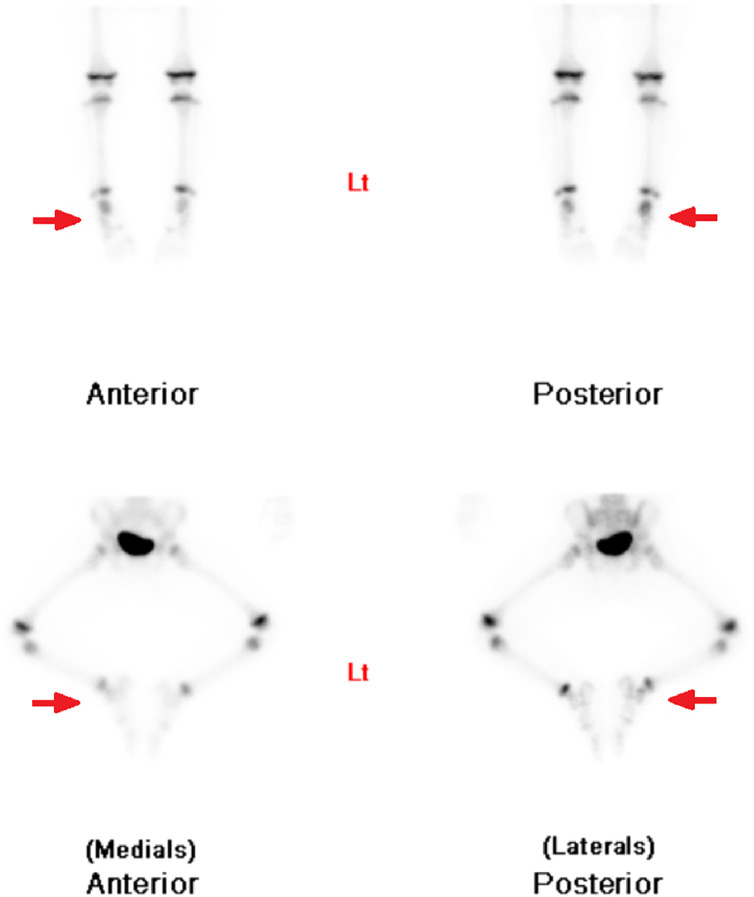
Delayed planar imaging of the feet, showing focal increased radiotracer uptake slightly inferior to growth plates of the right ankle.

**Fig. 3:**
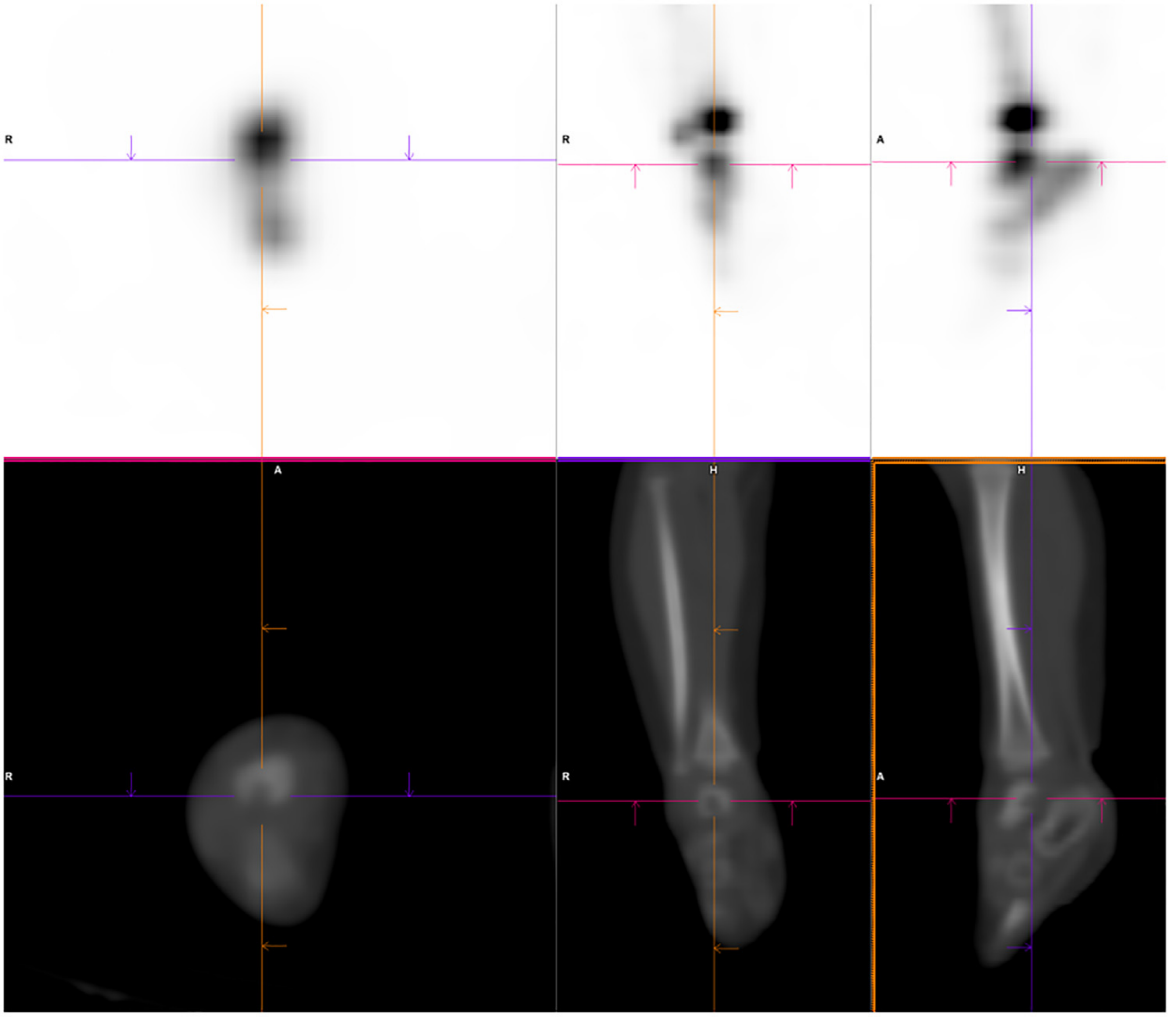
SPECT/CT imaging of the feet (axial, sagittal and coronal views), showing focal increased radiotracer uptake at the site of a well-defined lucent lesion in the posterior aspect of the talus of the right foot.

A triple-phase whole body bone scan was performed with the radiopharmaceutical ^99m^Technetium (^99m^Tc)-hydroxydiphosphonate (HDP). It demonstrated mild hyperemia at the right ankle, inferior to the growth plates, indicating an inflammatory process in this region (Fig. 1). Whole body and regional planar imaging (Fig. 2), and localized SPECT/CT imaging of the feet/ankles (Fig. 3) showed focal mild to moderately increased radiotracer uptake in the talus of the right foot, corresponding to a well-defined lucent lesion (or erosion) in the cortex at the posterior aspect of the talus on the low-dose CT. In addition, there was increased soft tissue density adjacent to the right talus, which was suggestive of surrounding edema.

**Fig. 4:**
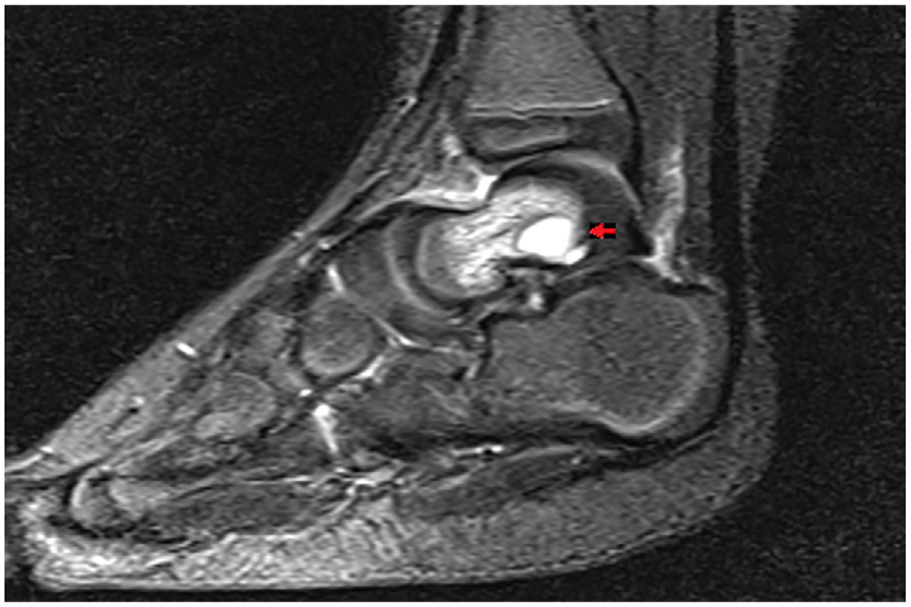
MRI STIR sequence imaging of the right foot, showing abnormal focal and diffuse hyper-intense signal changes in the posterior aspect of the talus.

Following the bone scan, an MRI of the right foot was performed. It demonstrated a 6 × 3 × 5 mm area of T2 and STIR hyperintensity in the posterior body of the talus (Fig. 4), which was in keeping with necrosis or fluid accumulation. There was evidence of adjacent marrow edema and fluid around the talus. The MRI findings were considered indicative of osteomyelitis and confirmed the bone scan findings.

The patient was commenced on intravenous cefazolin 50 mg/kg 3 times daily. She tolerated the antibiotics well, and after 48 hours she was transitioned to oral cephalexin and discharged with a plan to complete 6 weeks of antibiotic therapy. At the time of discharge, she was walking but still with her right foot on tiptoes, indicating incomplete resolution of symptoms. Follow-up was arranged in the outpatient orthopedic clinic.

### Case report #2

A 14-month-old male was transferred from an outside hospital for orthopedic admission due to a 5-day history of refusing to weight-bear on both legs. This was preceded by a 1-week history of flu-like symptoms and lethargy. On assessment, he was afebrile. His past medical history was unremarkable, and his immunizations were up to date. He was meeting all developmental milestones.

Initial investigations showed a normal WCC of 11 × 10^9^/L (normal range 4.5-15 × 10^9^/L) and mildly elevated CRP of 25 mg/L (normal range <3 mg/L). Musculoskeletal examination revealed he was unwilling to weight-bear. Passive and active range of motion of the bilateral hip, knee, and ankle joints was unremarkable, and there was no sacroiliac joint or lumbar spine tenderness. Ultrasound examination of both hip and knees joints did not identify an abnormality.

**Fig. 5:**
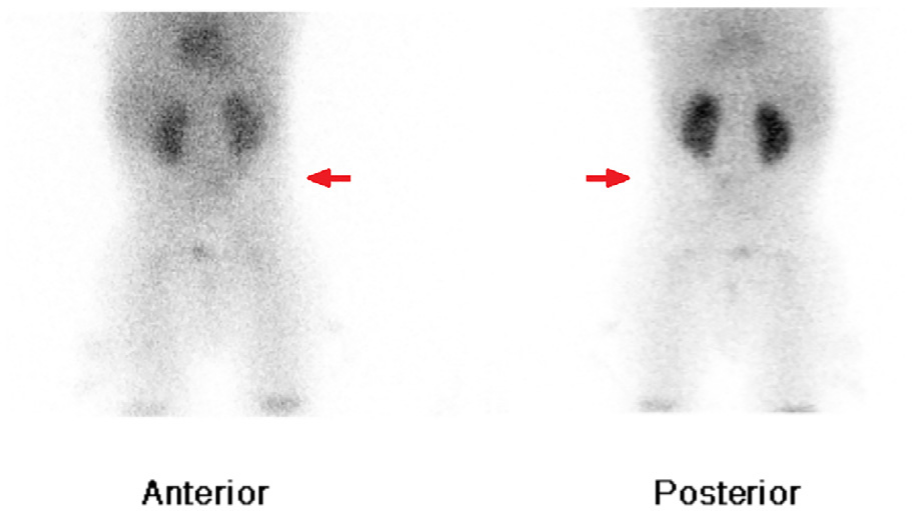
Blood-pool imaging from mid-chest to the knees, showing soft tissue hyperemia in the left aspect of the L5 vertebral body.

**Fig. 6:**
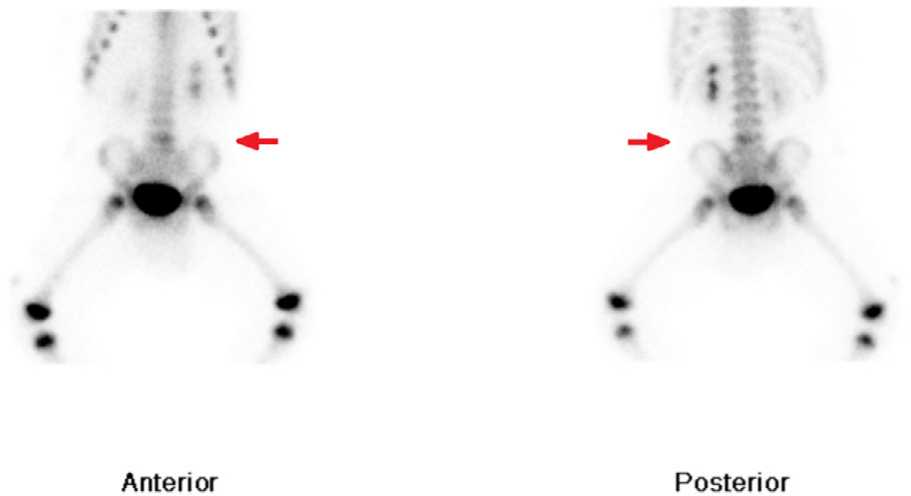
Delayed planar imaging of the mid-chest to the knees, showing focal increased radiotracer uptake in the left lower lumbar spine.

**Fig. 7:**
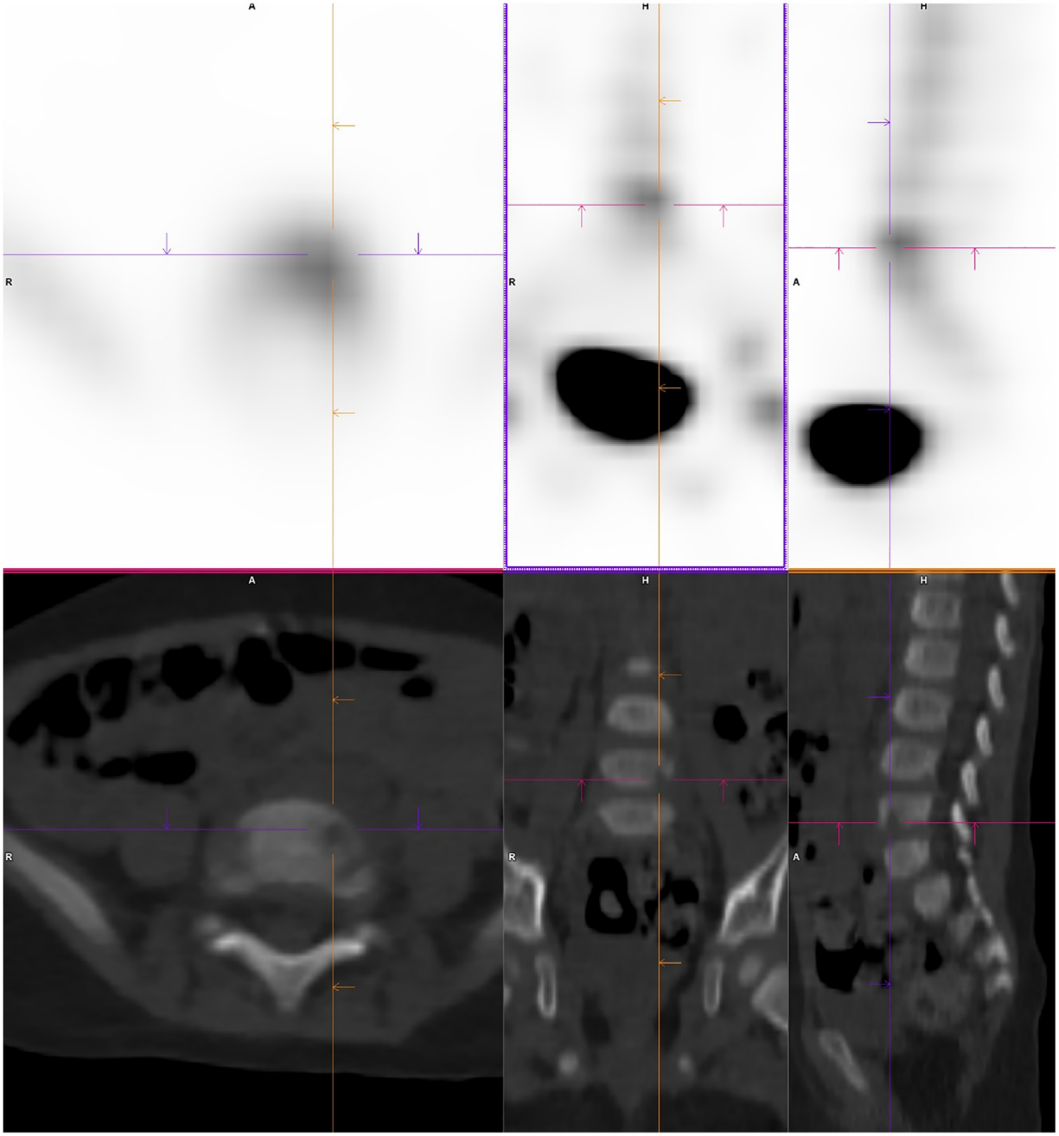
SPECT/CT imaging of the lumbar spine, showing focal increased radiotracer uptake at the site of bony erosion in the left aspect of the L5 vertebral body.

A triple-phase whole body bone scan with ^99m^Tc-HDP demonstrated mild focal hyperemia in the left side of the lower lumbar spine (Fig. 5). This correlated with increased focal radiotracer uptake in the left aspect of the L5 vertebral body on delayed planar imaging (Fig. 6) and SPECT/CT (Fig. 7). The concurrent low-dose CT identified the presence of a vertebral erosion at this site. The bone scan findings were diagnostic for osteomyelitis in the left aspect of the L5 vertebral body. Infectious diseases consultation suggested probable *Kingella kingae* osteomyelitis given the preceding upper respiratory tract symptoms, noting that a nasopharyngeal swab was positive for rhinovirus/enterovirus. The patient was commenced on intravenous antibiotics with a plan for a minimum of 2 weeks, followed by a step-down to oral antibiotics for a total of 6 weeks of treatment.

## Discussion

These cases illustrate the diagnostic challenges of identifying pediatric osteomyelitis. The first case presented with minimal systemic signs and normal inflammatory markers, making diagnosis particularly difficult. The second case, while presenting with fever and mildly elevated inflammatory markers, had nonspecific symptoms of refusal to walk, and initial examinations did not clearly localize the site of infection to the lumbar spine.

In the first case, plain radiographs were nondiagnostic. While radiographic latency is a known factor, the complex bony anatomy and osseous superimposition of the midfoot can also obscure subtle erosions in the talus, limiting the sensitivity of plain film even in subacute presentations [6]. The subsequent choice of imaging modality requires a balance between diagnostic sensitivity and procedural feasibility. Although MRI is the reference standard for soft tissue resolution and identifying marrow edema [7], the requirement for absolute immobility in children aged 1 to 4 years often necessitates GA, thereby introducing anesthetic risk and logistical delays [11]. Conversely, the quieter operation of SPECT/CT permits the use of accessible oral sedation protocols (eg, chloral hydrate), effectively removing the need for general anesthesia [12]. Furthermore, the literature supports the high diagnostic accuracy of SPECT/CT, with a reported sensitivity of 96% and specificity of 92% [13]. These findings demonstrate this modality as a sufficient diagnostic alternative, particularly when MRI is logistically challenging.

The diagnostic utility of SPECT/CT was demonstrated in the 2 presented cases. In the first case, SPECT/CT provided definitive diagnostic evidence by identifying focal increased radiotracer uptake corresponding to a region of cortical erosions. This structural finding confirmed established osteitis, adding specificity to the diagnosis beyond what is typically offered by visualizing marrow signal change on MRI. In the second case, the scan localized vertebral osteomyelitis despite the patient's poorly defined symptoms. A distinct advantage of bone scintigraphy in this demographic is the inherent whole-body survey; for nonverbal children with poorly localized pain (as in Case 2), this circumvents the field-of-view limitations associated with focused MRI [7]. Consequently, MRI was deemed unnecessary in the second case, as the SPECT/CT findings provided sufficient diagnostic confidence to direct treatment.

## Conclusion

These 2 cases demonstrate the value of triple-phase bone scintigraphy with SPECT/CT in diagnosing osteomyelitis in complex anatomical locations (talar bone and lumbar spine), particularly when the clinical presentation is equivocal. While diagnosis was complicated by normal inflammatory markers in the first case and nonlocalizing symptoms in the second, SPECT/CT provided precise anatomical localization of the infective focus by demonstrating increased tracer uptake and identifying subtle cortical erosions. This diagnostic accuracy was achieved without the logistical delays and risks associated with General Anesthesia often required for MRI. Therefore, ^99m^Tc-HDP SPECT/CT stands as a robust, alternative diagnostic tool for the timely evaluation of suspected osteomyelitis.

## Patient consent

Personal patient information was removed from this report. Informed written consent was obtained by the author for all the included cases.
